# Supplementary material for: Sequence-dependent nucleosome sliding in rotation-coupled and uncoupled modes revealed by molecular simulations
Source: PLoS Comput Biol. 2017 Dec 1;13(12):e1005880. doi: 10.1371/journal.pcbi.1005880 (PMC5728581; doi:10.1371/journal.pcbi.1005880)
Supplement: S1 Text — In the first section of the Supporting Information text we describe in detail the additional MD simulations of nucleosome repositioning via the loop-recapture mechanism and we compare the timescales of 601 repositioning via the three possible sliding modes (rotation-coupled, rotation-uncoupled and loop) using a set of 3-state kinetic models. In section 2 we describe the potential role of twist defects during rotation-coupled sliding. In section 3 we evaluate the dynamics of inserted arginine side chains during rotation-uncoupled sliding by generating all-atom models back-mapped from our coarse-grained trajectories. (PDF) [file pcbi.1005880.s012.pdf]

Sequence-dependent nucleosome sliding in rotation-coupled and uncoupled modes revealed by molecular simulations - supplementary information

Toru Niina, Giovanni B. Brandani, Cheng Tan, Shoji Takada

Department of Biophysics, Graduate School of Science, Kyoto University, Kyoto 606-8502, Japan

1 Sliding via the loop-recapture mechanism

In our unbiased MD simulations, we find that 601 nucleosomes slide via a rotation-uncoupled motion where the DNA moves by 5 bp at all contact points roughly simultaneously; repositioning to a 10-bp-shifted state similar to the crystal structure configuration is then completed after an analogous motion by a further 5 bp (SI Fig 1A-B). This is in contrast to the proposed mechanism of nucleosome sliding via DNA reptation.[11] According to this theoretical model, partial nucleosome unwrapping and DNA recapture can occasionally cause the formation of a loop defect at a nucleosome end. The size of this loop could vary, but it will be an integer of the DNA helical repeat length of ∼10 bp. In most cases, the loop will quickly dissipate to the closest nucleosome end, returning to the original nucleosome conformation. However, in some others cases the defect may diffuse around the nucleosome and be absorbed at the opposite nucleosome end, causing repositioning by a number of bp equal to the size of the loop (at least ∼10 bp). Using a version of Cafemol[7] patched with the software PLUMED,[12] we performed enhanced sampling MD simulations to investigate the possible role of DNA reptation in nucleosome sliding, and understand the causes of its absence in our unbiased simulations. In order to evaluate the free energy cost for the formation of a loop defect, we biased the system using well-tempered metadynamics[2] along two collective variables: N_X, the number of contacts between base pairs 30 to 70 (starting from the dyad) and the histone residues around super-helical locations (SHL) from 3.5 to 6.5; and N_{loop}, the number of contacts between base pairs 60 to 80 and the histone residues around SHL from 5.5 to 6.5. The configurations with large N_X and small N_{loop} correspond to the standard crystal structure form; whereas, conversely, the configurations with small N_X and large N_{loop} correspond to a configuration with a loop defect with a size of 10 bp. During the simulation, we also constrained the histone-DNA contacts up to SHL 2.5 to the native values, so that the loop defect is accommodated around SHL 4 and during the free-energy calculation it cannot diffuse to the opposite nucleosome end. To compute the number of histone-DNA contacts we employed the "coordination number" collective variable implemented in PLUMED with the default settings and a cutoff of 1.2 nm. Well-tempered metadynamics was performed with the following settings: bias factor = 20, hill deposition frequency = 500 MD steps, starting hill height = 1 kJ/mol, Gaussian hill widths = 20 and 10 for N_X and N_{loop} respectively. To provide an easier interpretation of the results, we reweighted[3] the free-energy surface along a single coordinate corresponding to the size of the loop defect ΔR, defined as the increase in the distance between base pair 45 and the center of mass of the octamer relative to the reference 3LZ0 crystal structure value. The free-energy profile, in SI Fig 2, shows that the crystal structure configuration lies in a deep free-energy minimum, and the formation of a loop defects has an energy cost of about 15 k_BT (evaluated from the probability of a loop with ΔR greater than 1 nm).

In order to estimate and compare the characteristic repositioning time scales of 601 nucleosomes via the three possible routes, we employed a 3-state master-equation model where the initial state corresponds to the 3LZ0 crystal structure, the final state to an equivalent conformation shifted by 10 bp, and the intermediate state to the 5-bp-shifted conformation observed in our unbiased simulations, an ideal 5-bp-rotated conformation generated from the reference crystal or the loop-defect conformation observed from metadynamics (SI Fig 3). These three intermediate states corresponds respectively to the rotation-uncoupled, rotation-coupled and loop-defect repositioning mechanisms. The average time to reposition by 10 bp can then be found from the lowest eigenvalue of the master equation of the 3-state system with an absorbing boundary condition at the final state, giving $T \simeq T_{0\to i}(T_{i\to 0}+T_{i\to 1})/T_{i\to 0}$, where 0, i and 1 correspond respectively to the initial, intermediate and final states (this approximate result holds when the rate from 0 to i is much smaller than all other rates, which is satisfied in all our cases). The inverse rates $T_{a\to b}$ (given in SI Fig 3) were estimated directly from unbiased MD from the mean first passage times to go from a to b, or from the detailed balance condition $p_a/p_b = T_{a\to b}/T_{b\to a}$, with $p_a/p_b = e^{-(F_a-F_b)/k_BT}$; the latter was employed to estimate $T_{0\to i}$ for the transitions from the crystal structure to the 5-bp-rotated and loop-defect conformations, with the free energy differences equal to 10 k_BT (as obtained by Freeman et al.[5]) and 15 k_BT (from metadynamics) respectively. Altogether, we find that the times to reposition by 10 bp are $\sim$2.4x10^9, $\sim$1.5x10^{11}, $\sim$2.2x10^{13} MD steps for the rotation-uncoupled, rotation-coupled and

loop-defect routes respectively. Therefore, for 601 nucleosomes, the rotation-uncoupled mode via the intermediate 5-bp-shifted state, observed from unbiased MD, highly dominates over the other two possible mechanisms. In particular, the DNA reptation via loop defect propagation is expected to play the smallest role in nucleosome repositioning, due to the high free-energy cost to form an initial loop defect in the first place, but also to the high probability that the loop will immediately escape towards the closest nucleosome end, instead of diffusing to the opposite end causing sliding. For higher hydrogen bond strengths, the rotation-coupled mode should eventually dominate over the other two, since this is the only mechanism where the intermediate states preserve all histone-DNA contacts of the nucleosome (see also the discussion in the main text).

2 Twist defects during rotation-coupled sliding

The rotation-coupled motion of DNA observed during the repositioning of polyCG nucleosomes is consistent with the proposed theory of sliding via twist defect propagation.[9] Twist defects are small deformations of the DNA path that allow the nucleosome to accommodate different numbers of base pairs between two neighboring histone-DNA contact points. The screw-like motion of DNA causing repositioning should be facilitated by the formation of these twist defects, since they would allow histone-DNA interactions at the different contact points to be broken only one at the time. To investigate the presence of these defects in our simulations we plotted in SI Fig 4 the motion of DNA at the super-helical locations corresponding to the 10 central nucleosome contact points (at the further and weaker contact points data are very noisy). These time-lines show that DNA motion is mostly cooperative, except around SHL ± 2, where the motion of DNA at the neighboring contact points (SHL ± 2.5 and ± 1.5) seems to suggest the formation of a twist defect corresponding to a missing base pair at these locations. However, our data are too noisy to make conclusive statements about the role of these defects in the repositioning kinetics, and we plan to study these features in more details in the future.

3 Role of inserted Arginines

In the nucleosome there are many arginine side chains inserted into the DNA minor grooves, and their role in inhibiting nucleosome sliding has been documented.[4] Even though arginine-DNA interactions are included via the long-range electrostatics and hydrogen-bonding terms, in our model each residue is represented via a single bead centered at the C_α atom, and the specific steric effects due to side chains are not explicitly modeled. In our MD simulations of 601 nucleosomes, we observed repositioning via a rotation-uncoupled mode where the DNA moves similarly to a rigid body relative to the histone octamer. One may suspect that a more appropriate modeling of the inserted arginines would completely prevent this type of motion. To investigate this possibility, we took several configurations observed during a transition between the crystal form and the 5-bp-shifted state (SI Fig 5A), and back-mapped the coarse-grained coordinates to generate a fully atomistic model of the transition. The DNA coordinates were generated by aligning each base pair as observed in the 3LZ0 crystal structure to the corresponding coarse-grained beads in the structures from MD. For the protein part, the backbone was modeled using the "PD2 ca2main" web-server,[10] whereas for the side chains we employed SCWRL4.[8] The nucleosome was then solvated in water, the charge was neutralized with a minimum number of sodium ions, and the resulting structure was relaxed via energy minimization and 100 ps of MD simulation in the NVT ensemble using the AMBER Parmbsc1 force field[6] and the software GROMACS 5.[1] As we can see from SI Fig 5C-E, a simple alignment of the rigid histone octamer structure to the coarse-grained coordinates, without relaxation, causes clashes between inserted arginines and DNA backbone during the rotation-uncoupled motion of DNA. On the other hand, an appropriate modeling of side chains and MD relaxation (as described) avoids this problem. In particular, the modeling shows that during the transition, side chains could exit the DNA minor groove and allow the DNA rotation-uncoupled movement. Therefore, while an accurate modeling of arginine side chains could correctly describe their inhibition of nucleosome sliding,[4] side-chain fluctuations should still permit the repositioning mechanism observed in our MD simulations.

References

[1] M. J. Abraham, T. Murtola, R. Schulz, S. Páll, J. C. Smith, B. Hess, and E. Lindahl. Gromacs: High performance molecular simulations through multi-level parallelism from laptops to supercomputers. *SoftwareX*, 1:19–25, 2015.

[2] A. Barducci, G. Bussi, and M. Parrinello. Well-tempered metadynamics: a smoothly converging and tunable free-energy method. *Physical review letters*, 100(2):020603, 2008.

[3] M. Bonomi, A. Barducci, and M. Parrinello. Reconstructing the equilibrium boltzmann distribution from well-tempered metadynamics. *Journal of computational chemistry*, 30(11):1615–1621, 2009.

[4] A. Flaus, C. Rencurel, H. Ferreira, N. Wiechens, and T. Owen-Hughes. Sin mutations alter inherent nucleosome mobility. *The EMBO journal*, 23(2):343–353, 2004.

[5] G. S. Freeman, J. P. Lequieu, D. M. Hinckley, J. K. Whitmer, and J. J. de Pablo. Dna shape dominates sequence affinity in nucleosome formation. *Physical review letters*, 113(16):168101, 2014.

[6] I. Ivani, P. D. Dans, A. Noy, A. Pérez, I. Faustino, A. Hospital, J. Walther, P. Andrio, R. Goñi, A. Balaceanu, et al. Parmbsc1: a refined force-field for dna simulations. *Nature methods*, 13(1):55, 2016.

[7] H. Kenzaki, N. Koga, N. Hori, R. Kanada, W. Li, K.-i. Okazaki, X.-Q. Yao, and S. Takada. Cafemol: a coarse-grained biomolecular simulator for simulating proteins at work. *Journal of Chemical Theory and Computation*, 7(6):1979–1989, 2011.

[8] G. G. Krivov, M. V. Shapovalov, and R. L. Dunbrack. Improved prediction of protein side-chain conformations with scwrl4. *Proteins: Structure, Function, and Bioinformatics*, 77(4):778–795, 2009.

[9] I. Kulić and H. Schiessel. Chromatin dynamics: nucleosomes go mobile through twist defects. *Physical review letters*, 91(14):148103, 2003.

[10] B. L. Moore, L. A. Kelley, J. Barber, J. W. Murray, and J. T. MacDonald. High–quality protein backbone reconstruction from alpha carbons using gaussian mixture models. *Journal of computational chemistry*, 34(22):1881–1889, 2013.

[11] H. Schiessel, J. Widom, R. Bruinsma, and W. Gelbart. Polymer reptation and nucleosome repositioning. *Physical review letters*, 86(19):4414, 2001.

[12] G. A. Tribello, M. Bonomi, D. Branduardi, C. Camilloni, and G. Bussi. Plumed 2: New feathers for an old bird. *Computer Physics Communications*, 185(2):604–613, 2014.
